# Supplementary material for: Spatial and temporal dynamics at an actively silicifying hydrothermal system
Source: Front Microbiol. 2023 May 3;14:1172798. doi: 10.3389/fmicb.2023.1172798 (PMC10188993; doi:10.3389/fmicb.2023.1172798)
Supplement: Supplementary file 1 [file Data_Sheet_1.PDF]

Supplementary Material

1 Supplementary Tables

Supplementary Table 1. Sampling summary for major types of analysis/quantification for each sampling time point. Dates are shown as year-month-day.

Sampling Date	2010-6-4	2017-8-18	2018-5-30	2018-7-26	2018-9-29	2019-8-9	2020-8-21
**16S rRNA Gene**	x	x	x	x	x	x	x
**16S rRNA**	-	-	-	-	x	x	x
**IC**	-	x	x	x	x	x	x
**ICP**	-	x	x	x	x	x	x
**pH**	-	x	x	x	x	x	x
**Temperature**	-	x	x	x	x	x	x
**DIC/DOC**	-	-	-	x	x	x	x
**Notes**	No samples at 0m	Only sampled spring source		No DIC/DOC sample at 3m		DIC/DOC vials at 4 and 5m broke in transit	

Supplementary Table 2. Alpha diversity pairwise statistical tests results comparing group1 and group2 entries for each row, either by date or distance from spring source in meters. The Kruskal-Wallis and pairwise Wilcoxon tests were used to determine alpha diversity statistical significances (adjusted *p*-value < 0.05), along with the Wilcoxon effect size. Only statistically significant results are shown (adjusted *p* < 0.05). * denotes *p* < 0.05, ** denotes *p* < 0.005, *** denotes *p* < 0.0005, **** denotes *p* < 0.00005. Mag. denotes magnitude of effect size and stat denotes statistic

metric	category	group 1	group 2	n 1	n 2	stat	p	p.adj	p.adj. signif	effect size	mag.
**evenness**	date	4-Jun-10	18-Aug-17	14	2	28	0.017	0.044	*	0.556	large
**evenness**	date	4-Jun-10	30-May-18	14	16	185	0.002	0.01	*	0.554	large
**evenness**	date	4-Jun-10	21-Aug-20	14	16	196	2.3E-04	0.005	**	0.638	large
**evenness**	date	18-Aug-17	26-Jul-18	2	16	0	0.013	0.039	*	0.53	large
**evenness**	date	18-Aug-17	29-Sep-18	2	16	0	0.013	0.039	*	0.53	large
**evenness**	date	30-May-18	29-Sep-18	16	16	48	0.002	0.01	*	0.533	large
**evenness**	date	26-Jul-18	21-Aug-20	16	16	195	0.011	0.039	*	0.446	moderate
**evenness**	date	29-Sep-18	21-Aug-20	16	16	215	0.001	0.007	**	0.58	large
**evenness**	distance	0 meters	4 meters	7	18	14	0.002	0.019	*	0.593	large
**evenness**	distance	0 meters	5 meters	7	18	17	0.004	0.019	*	0.557	large
**evenness**	distance	0 meters	1 meters	7	17	23	0.02	0.042	*	0.473	moderate
**evenness**	distance	0 meters	2 meters	7	18	16	0.003	0.019	*	0.569	large
**evenness**	distance	0 meters	3 meters	7	18	19	0.006	0.023	*	0.533	large
**evenness**	distance	1 meters	4 meters	17	18	77	0.011	0.034	*	0.424	moderate
**evenness**	distance	1 meters	5 meters	17	18	81	0.017	0.042	*	0.402	moderate
**richness**	date	4-Jun-10	29-Sep-18	14	16	223	2.8E-08	5.78E-07	****	0.842	large
**richness**	date	30-May-18	29-Sep-18	16	16	246	4.6E-07	3.24E-06	****	0.786	large
**richness**	date	26-Jul-18	29-Sep-18	16	16	237	6.9E-06	3.62E-05	****	0.726	large
**richness**	date	26-Jul-18	21-Aug-20	16	16	47	0.002	0.006	**	0.54	large
**richness**	date	29-Sep-18	11-Aug-19	16	16	44	0.001	0.004	**	0.56	large
**richness**	date	29-Sep-18	21-Aug-20	16	16	9	3.2E-07	3.24E-06	****	0.793	large
**richness**	distance	0 meters	4 meters	7	18	21	0.009	0.035	*	0.508	large
**richness**	distance	0 meters	5 meters	7	18	14	0.002	0.009	**	0.593	large
**richness**	distance	1 meters	4 meters	17	18	56	0.001	0.007	**	0.541	large
**richness**	distance	1 meters	5 meters	17	18	48	3E-04	0.004	**	0.586	large

Supplementary Table 3. Beta diversity pairwise statistical test results. Permutational analysis of variance (PERMANOVA) and pairwise comparisons (adjusted *p*-value < 0.05) were calculated using Vegan to test for statistical significance between beta diversity groupings across dates and locations (10000 permutations). Only statistically significant results are shown (adjusted *p* < 0.05). Pairwise comparisons were performed between entries in variable 1 column and variable 2 column.

test variable	p.value	adj.p.value	variable 1	variable 2	sig	R2
**Distance from spring**	0.019	0.019	4 meters	3 meters	TRUE	0.066
**Distance from spring**	0.032	0.032	2 meters	3 meters	TRUE	0.065
**Sampling date**	1.00E-04	1.00E-04	4-Jun-10	30-May-18	TRUE	0.234
**Sampling date**	1.00E-04	1.00E-04	4-Jun-10	26-Jul-18	TRUE	0.277
**Sampling date**	1.00E-04	1.00E-04	4-Jun-10	29-Sep-18	TRUE	0.184
**Sampling date**	1.00E-04	1.00E-04	4-Jun-10	11-Aug-19	TRUE	0.183
**Sampling date**	3.00E-04	3.00E-04	4-Jun-10	21-Aug-20	TRUE	0.14
**Sampling date**	0.042	0.042	30-May-18	26-Jul-18	TRUE	0.073
**Sampling date**	2.00E-04	2.00E-04	30-May-18	11-Aug-19	TRUE	0.153
**Sampling date**	2.00E-04	2.00E-04	30-May-18	21-Aug-20	TRUE	0.134
**Sampling date**	3.00E-04	3.00E-04	26-Jul-18	11-Aug-19	TRUE	0.137
**Sampling date**	1.00E-04	1.00E-04	26-Jul-18	21-Aug-20	TRUE	0.197
**Sampling date**	0.011	0.011	29-Sep-18	21-Aug-20	TRUE	0.099
**Sampling date**	0.004	0.004	11-Aug-19	21-Aug-20	TRUE	0.125

2 Supplementary Figures

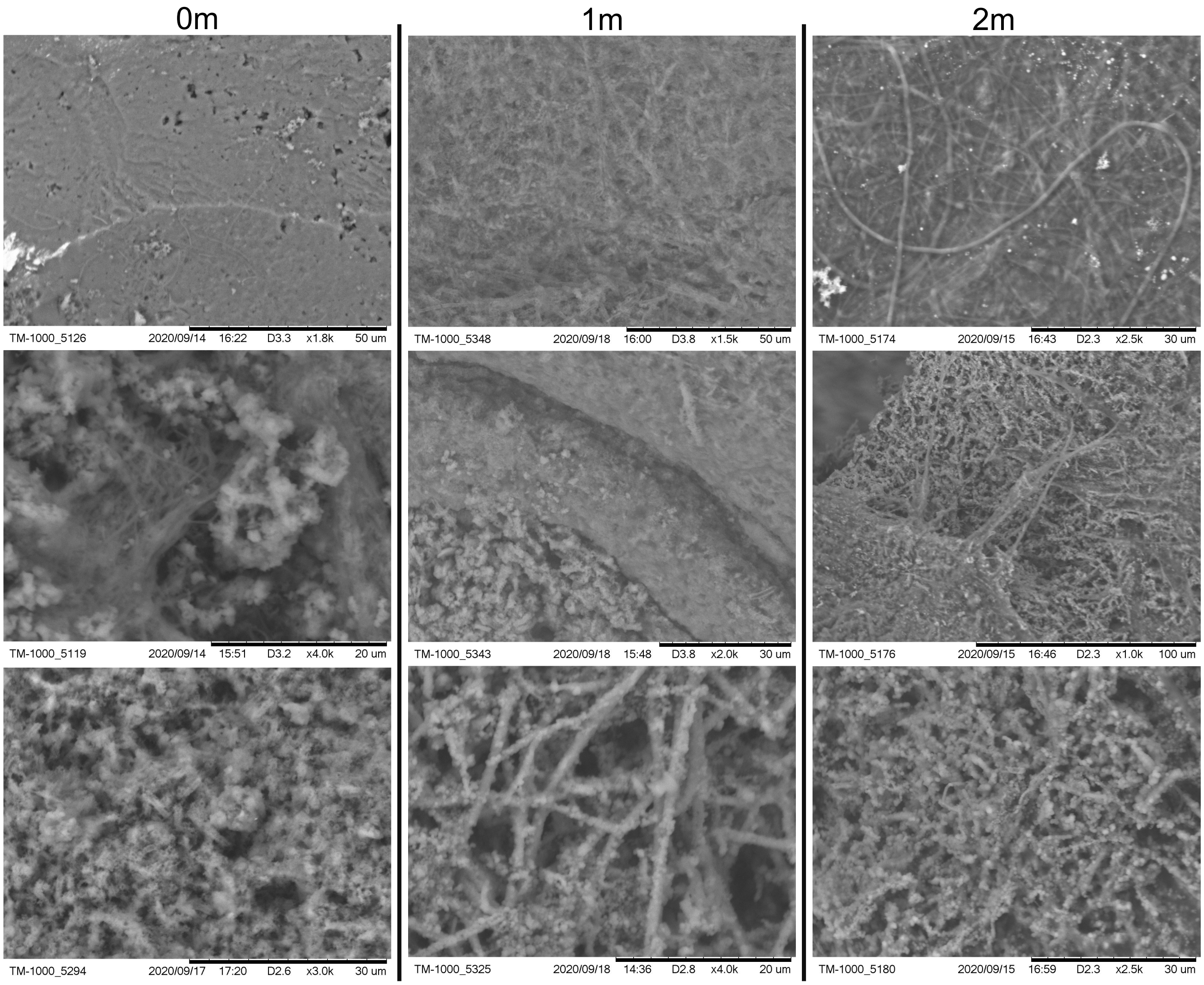

Supplementary Figure 1. Scanning electron microscope images taken of the Steep Cone Geyser outflow channel biofilm and underlying silicified matrix at 0, 1, and 2 meters. Top numbers indicate distance from the hydrothermal source the samples were taken at in meters. Each column corresponds to the listed distance. Images illustrate thin EPS and filament rich biofilm atop a fully silicified microbial matrix.

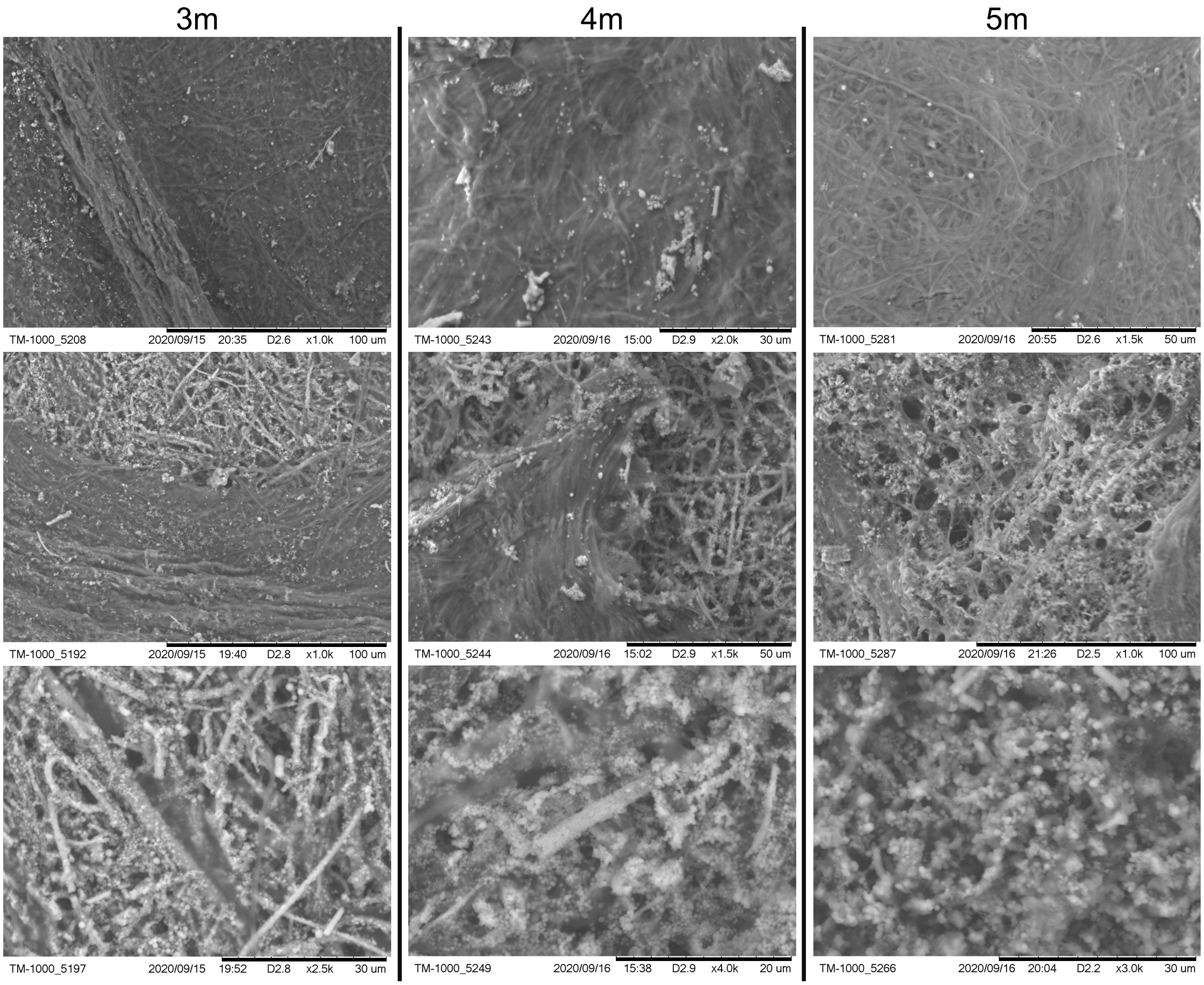

Supplementary Figure 2. Scanning electron microscope images taken of the Steep Cone Geyser outflow channel biofilm and underlying silicified matrix at 3, 4, and 5 meters. Top numbers indicate distance from the hydrothermal source the samples were taken at in meters. Each column corresponds to the listed distance. Images illustrate thin EPS and filament rich biofilm atop a fully silicified microbial matrix.

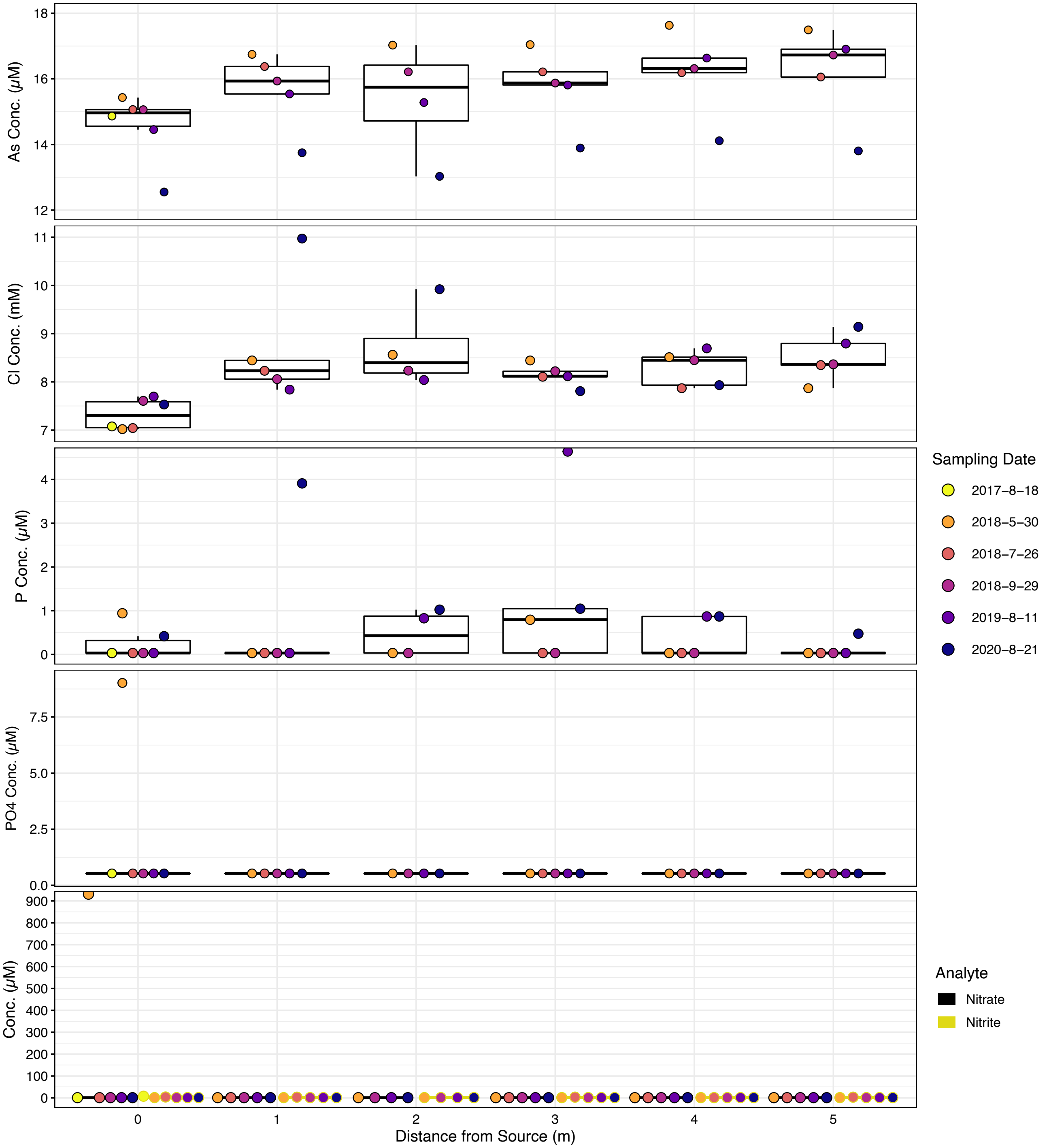

Supplementary Figure 3. Dot and box plot illustrating geochemical stability down the sampled transect across sampling dates of geochemical parameters of interest. Filled dot colors indicate sampling date while box colors indicate measured parameter. Dots are arranged from left to right, oldest to most recent samples respectively. Dates are shown as year-month-day.

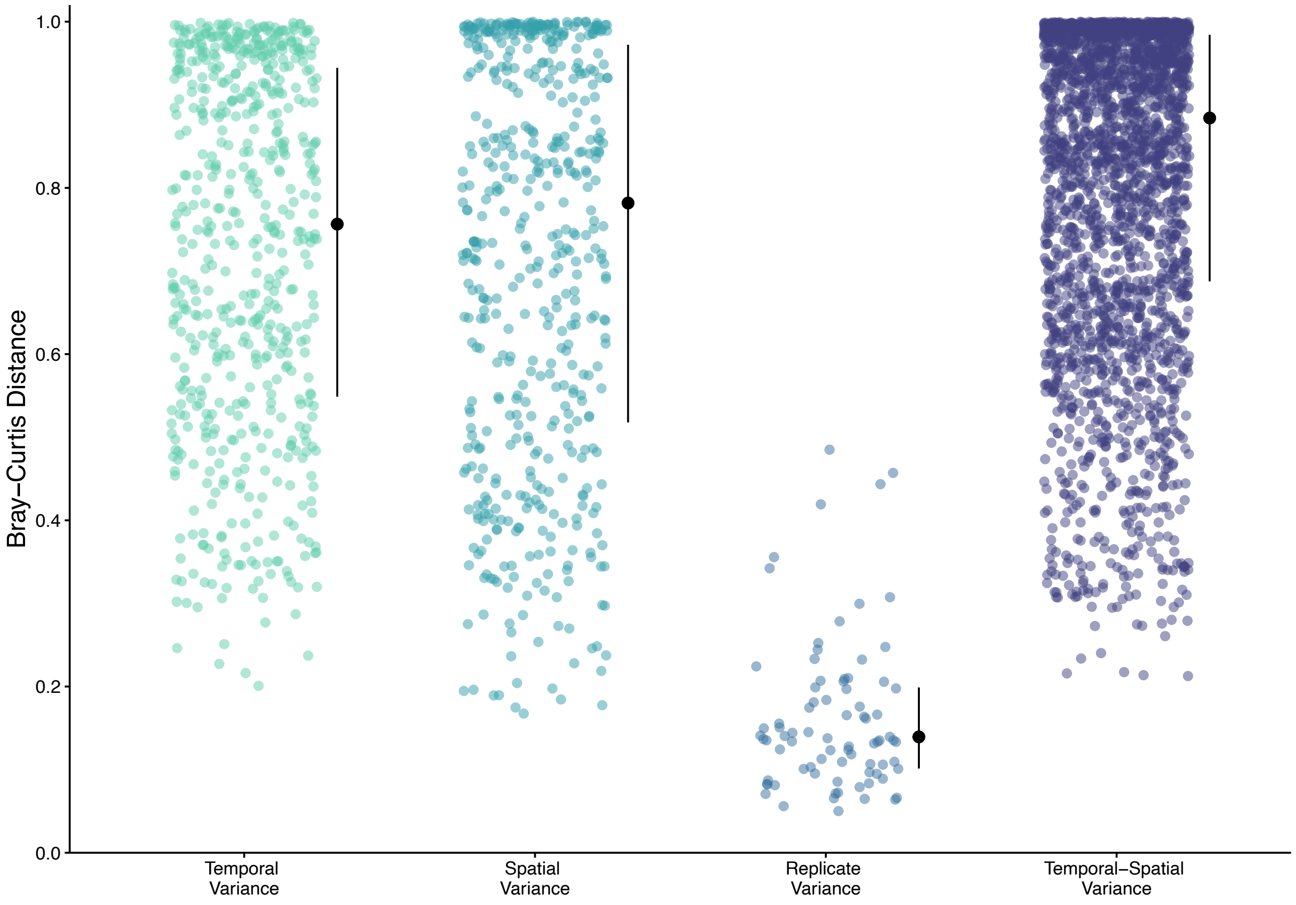

Supplementary Figure 4. Plot displaying the distribution of Bray-Curtis distances between samples. Colored dots represent ecological distances between samples. Black dots indicate the median, while black lines indicate the interquartile range (50% confidence interval).

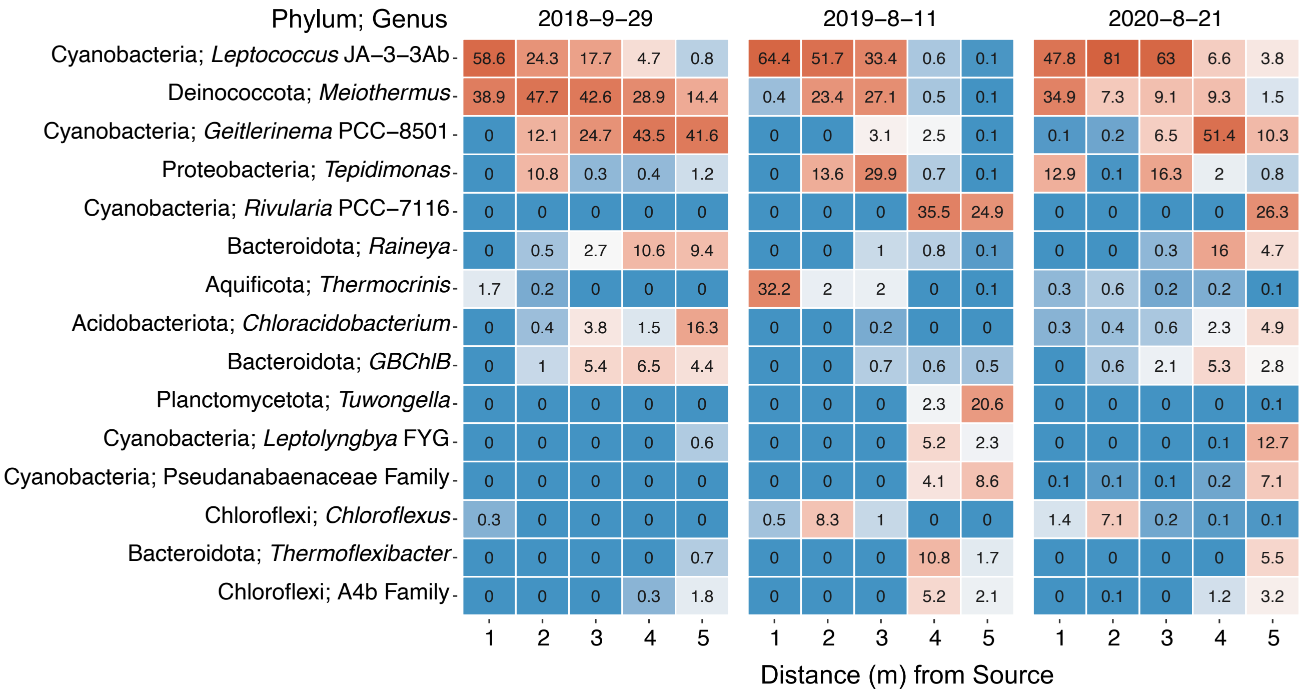

Supplementary Figure 5. Heat map of the top 15 taxa within the bacterial and archaeal communities from 16S rRNA sequencing. Taxa are named by phylum and genus or lowest classification. Values indicate the mean percent relative abundance. Data is faceted by sampling date and ordered down the sampling transect. Dates are shown as year-month-day.

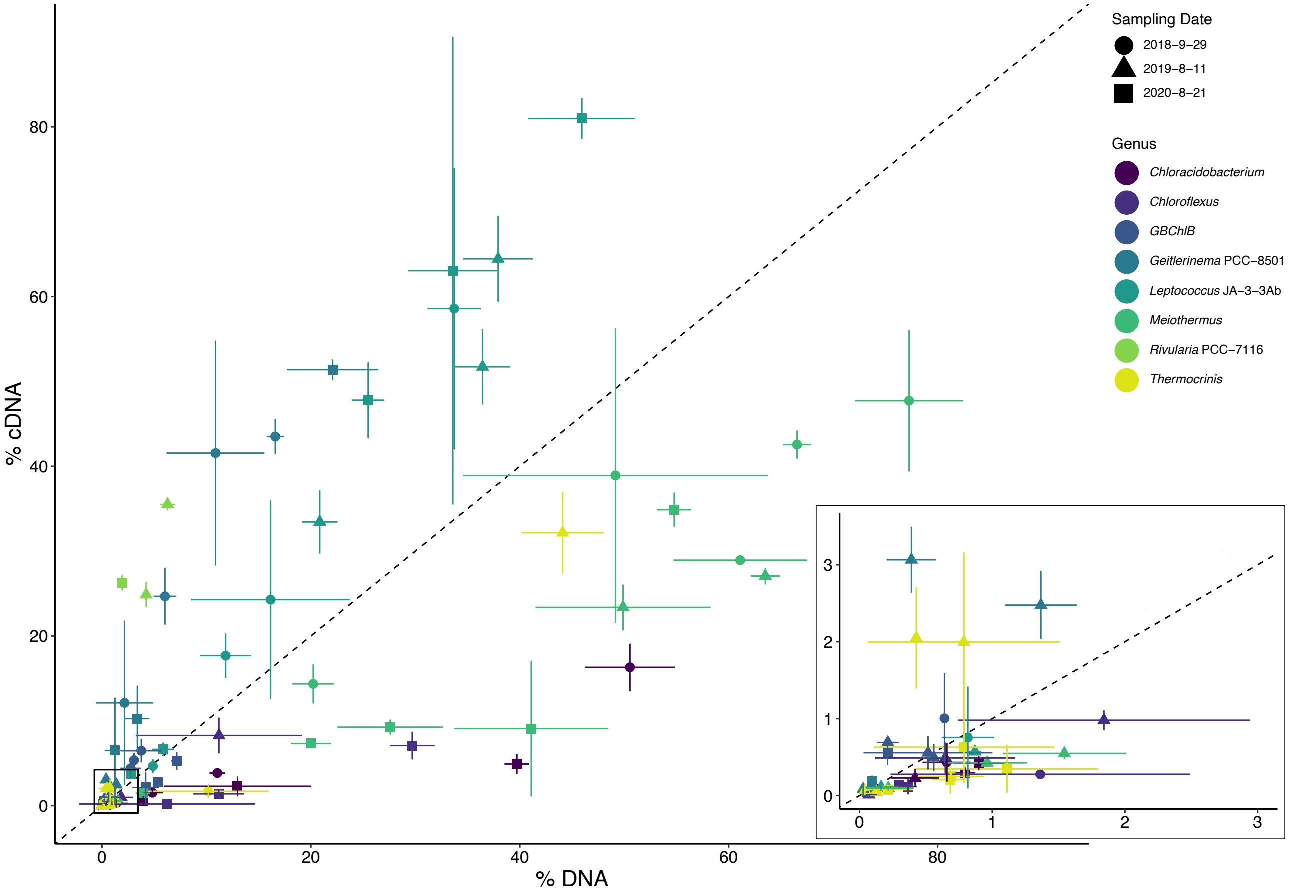

Supplementary Figure 6. Abundances of the top 8 genera as indicated by 16S rRNA sequence abundance. Inset of samples located within the black box of 0 to 3 % (c)DNA. Axes are percent relative abundance in either the cDNA (16S rRNA) or DNA (16S rRNA gene) sequencing data. Dots represent mean relative abundance and error bars indicate the standard deviation (n = 3) in the cDNA and DNA of biomass collected from the biofilms along the samples transect at Steep Cone Geyser. Genus classification is indicated by dot colors, while shapes indicate sampling date. Dotted line represents a 1:1 ratio of % cDNA to % DNA. Dates are shown as year-month-day.

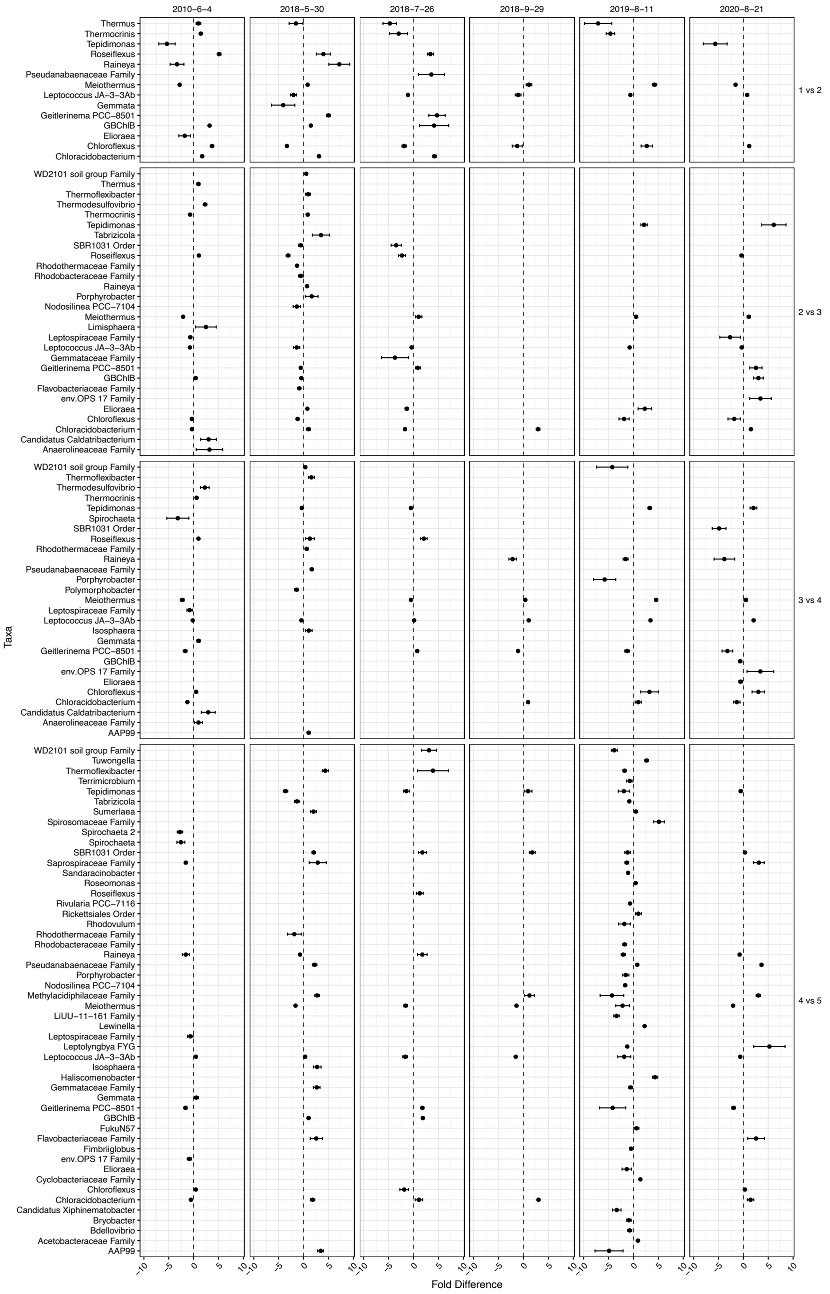

Supplementary Figure 7. Results from differential abundance analysis of 16S rRNA gene sequencing conducted using Corncob. Differential abundance comparison was conducted between adjacent sampling locations (right) for each sampling date (top). X-axis is Log_2 fold change. Listed taxa were determined to be statistically significant and are named by genus or lowest classification. All comparisons were performed sequentially. Therefore, the baseline comparison for each pairwise set is the lower number. Only taxa determined to be statistically significant for differential abundance after false discovery rate correction (*p-adj.* < 0.05) are shown. Positive values indicate the taxon increased in abundance from the baseline, while negative values indicate the taxon decreased in abundance from the baseline. Dates are shown as year-month-day.

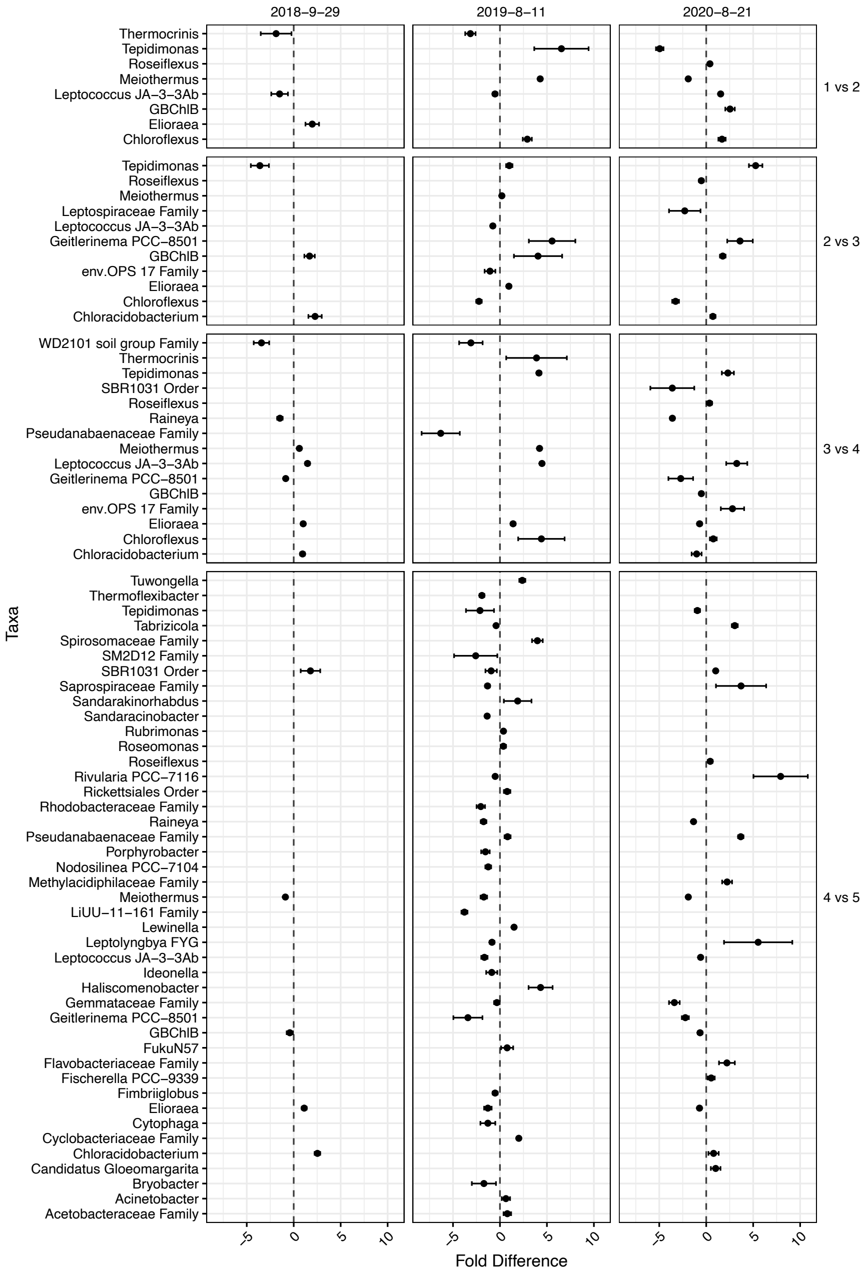

Supplementary Figure 8. Results from differential abundance analysis of 16S rRNA sequencing conducted using Corncob. Differential abundance comparison was conducted between adjacent sampling locations (right) for each sampling date (top). X-axis is Log_2 fold change. Listed taxa were determined to be statistically significant and are named by genus or lowest classification. All comparisons were performed sequentially. Therefore, the baseline comparison for each pairwise set is the lower number. Only taxa determined to be statistically significant for differential abundance after false discovery rate correction (*p-adj.* < 0.05) are shown. Positive values indicate the taxon increased in abundance from the baseline, while negative values indicate the taxon decreased in abundance from the baseline. Dates are shown as year-month-day.

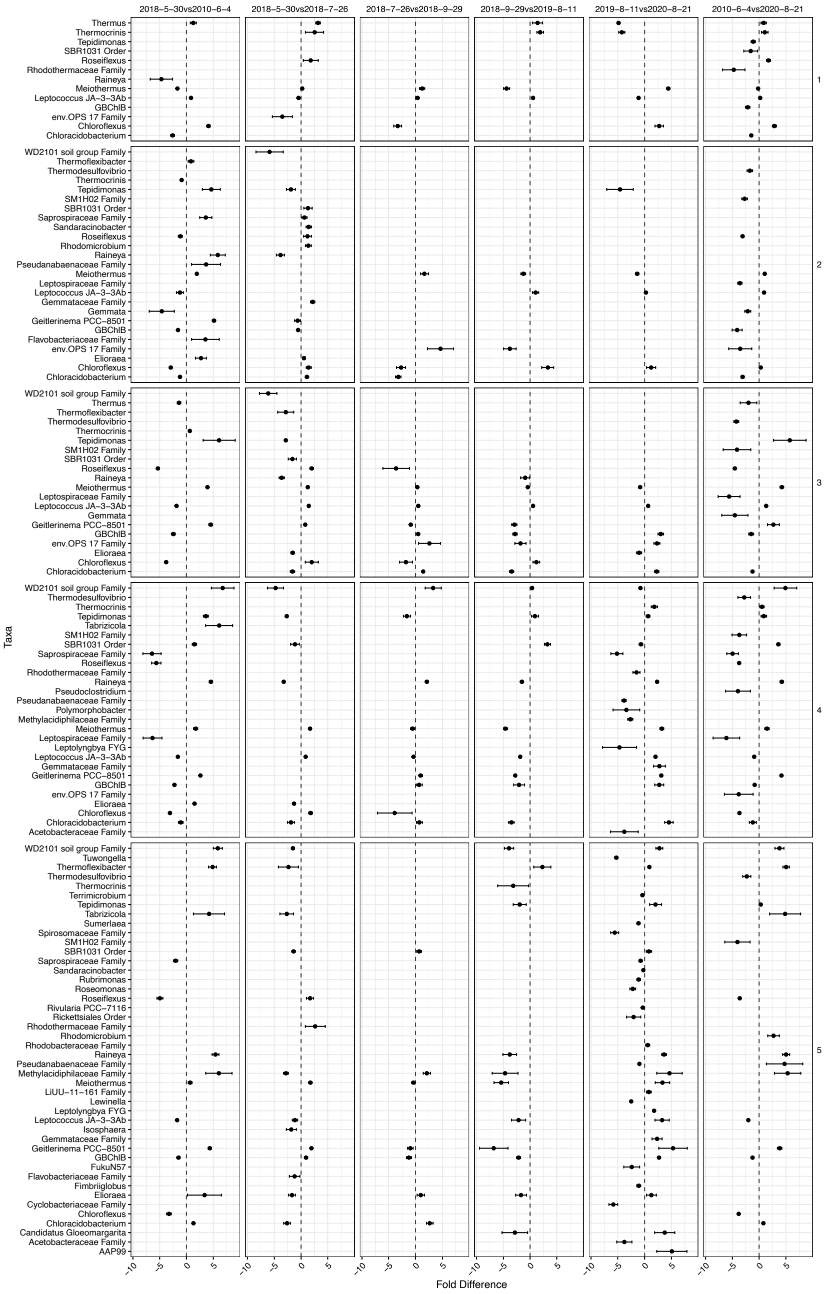

Supplementary Figure 9. Results from differential abundance analysis of 16S rRNA gene sequencing conducted using Corncob. Differential abundance comparison was conducted between sampling dates (top) for each sampling location (right). X-axis is Log_2 fold change. Listed taxa were determined to be statistically significant and are named by genus or lowest classification. All comparisons were performed sequentially. Therefore, the baseline comparison for each pairwise set is the lower number. Only taxa determined to be statistically significant for differential abundance after false discovery rate correction (*p-adj.* < 0.05) are shown. Positive values indicate the taxon increased in abundance from the baseline, while negative values indicate the taxon decreased in abundance from the baseline. Dates are shown as year-month-day.

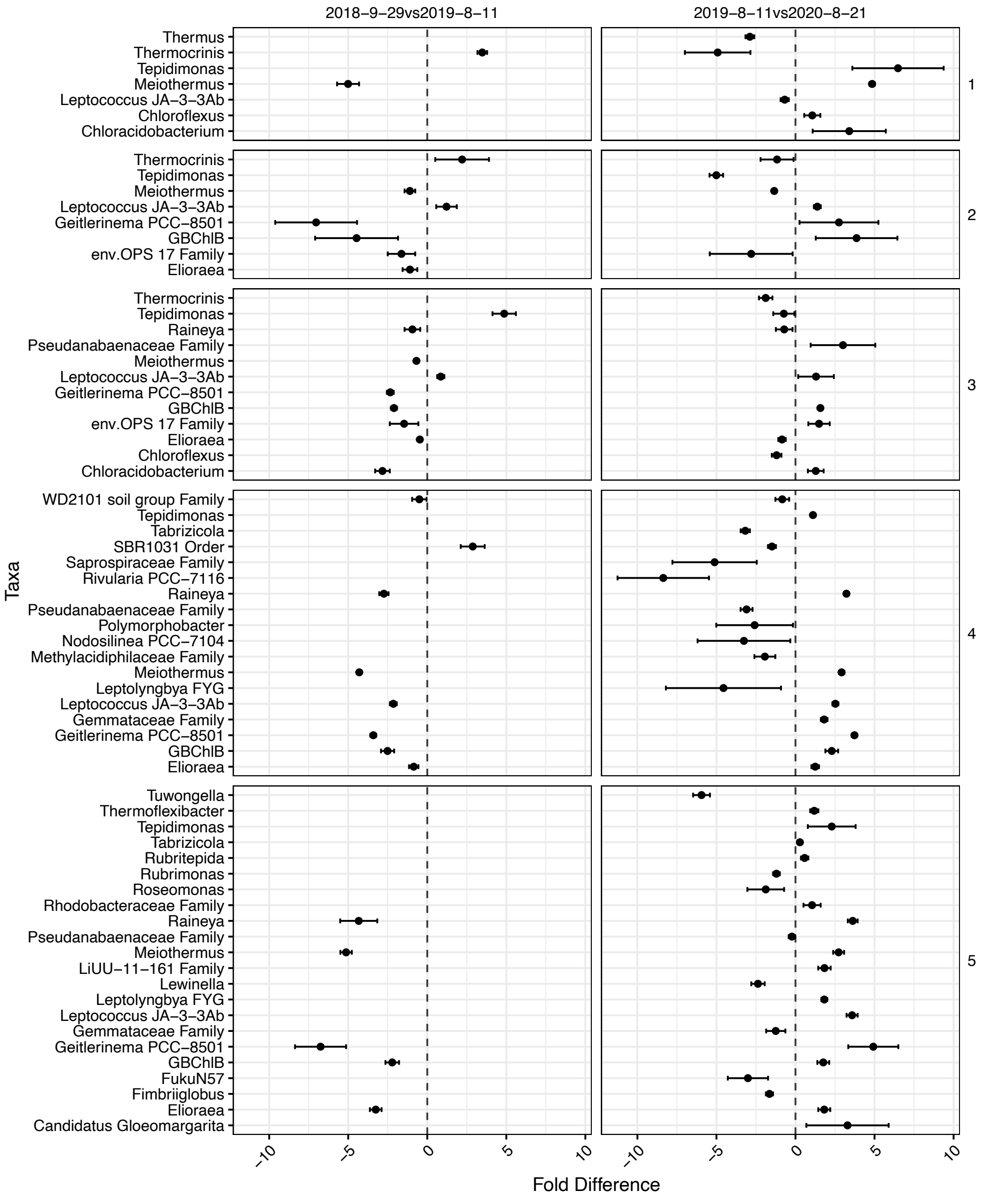

Supplementary Figure 10. Results from differential abundance analysis of 16S rRNA sequencing conducted using Corncob. Differential abundance comparison was conducted between sampling dates (top) for each sampling location (right). X-axis is Log_2 fold change. Listed taxa were determined to be statistically significant and are named by genus or lowest classification. All comparisons were performed sequentially. Therefore, the baseline comparison for each pairwise set is the lower number. Only taxa determined to be statistically significant for differential abundance after false discovery rate correction (*p-adj.* < 0.05) are shown. Positive values indicate the taxon increased in abundance from the baseline, while negative values indicate the taxon decreased in abundance from the baseline. Dates are shown as year-month-day.

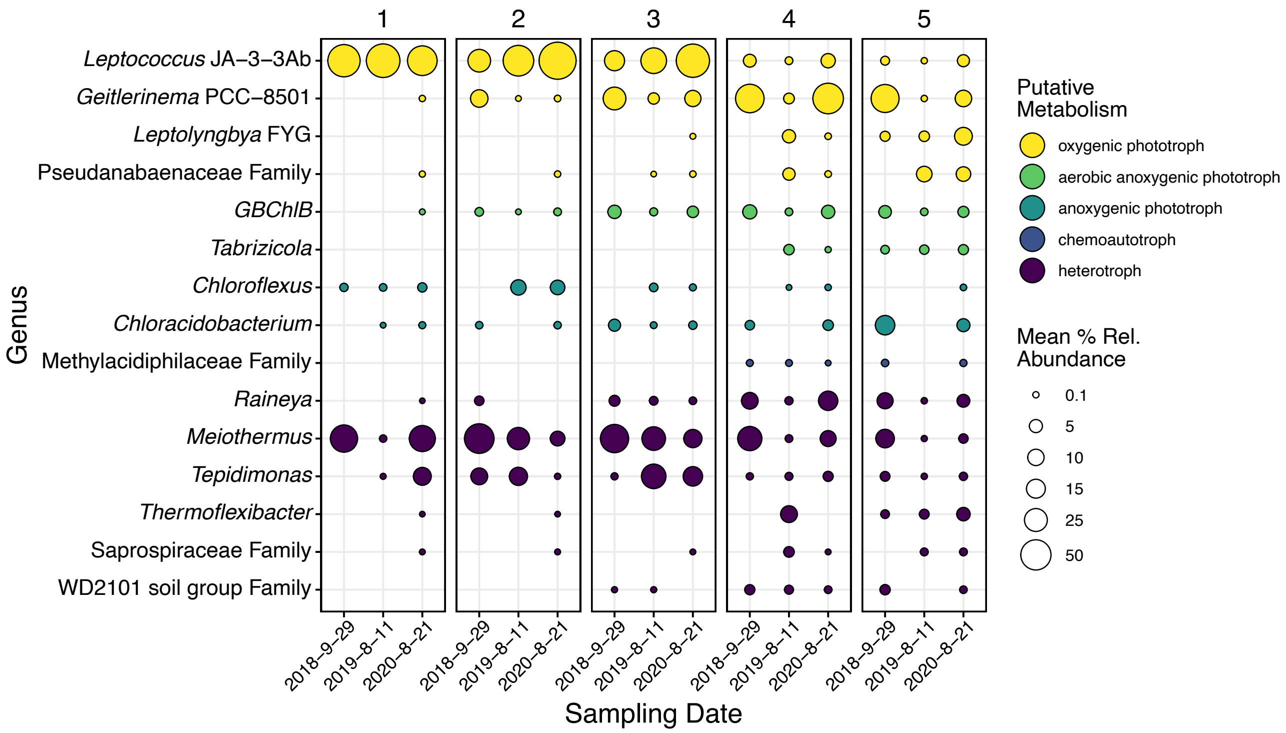

Supplementary Figure 11. 16S rRNA mean percent relative abundances of genera (or lowest classification) determined to be statistically significant by SIMPER, DNA differential abundance analysis, and cDNA differential abundance analysis. Panels are faceted by distance (m) from the hydrothermal source. Circle sizes indicate mean percent relative abundance. Colors indicate general putative metabolisms associated with the most abundant ASVs in each genus according to NCBI BLAST searches and literature review. Dates are shown as year-month-day.
